# Peutz-Jeghers Syndrome Presenting With Iron-Deficiency Anaemia and a Giant Colonic Polyp

**DOI:** 10.7759/cureus.78520

**Published:** 2025-02-04

**Authors:** Naznin Naher, Abdullah Al Masud, Sunil Kumar Biswas, Hasan IMAM, Md Nazmul Hasan

**Affiliations:** 1 Department of Internal Medicine, Bangabandhu Sheikh Mujib Medical University, Dhaka, BGD; 2 Internal Medicine, St. Louis University School of Medicine, Missouri, USA

**Keywords:** anaemia, cancer screening, giant colonic polyp, hamartomatous polyp, intestinal hamartoma

## Abstract

Peutz-Jeghers syndrome (PJS) is an uncommon autosomal dominant disorder that manifests as mucocutaneous pigmentation and hamartomatous polyps in the gastrointestinal system. Pigmentation of the skin and mucous membranes may be present from birth, but it typically appears in early childhood and can sometimes develop later. In addition to an increased lifelong risk of cancers and problems, such as gastrointestinal bleeding from polyposis, hamartomatous polyps can develop in the stomach, small bowel, or colon. In order to prevent difficulties associated with intestinal and extraintestinal malignancies in patients diagnosed with PJS, routine screening is essential. We report a case of a 19-year-old young girl who presented with intermittent episodes of abdominal pain and a few occasions of fresh per rectal bleeding, as well as features of anaemia.

## Introduction

Peutz-Jeghers syndrome (PJS), also known as periorificial lentiginosis, is a rare autosomal dominant disorder characterised by gastrointestinal polyps and mucocutaneous pigmentation. While pigmentation can appear later in life, it typically manifests in early childhood [1]. Hamartomatous polyps develop in the colon, small intestine, or stomach, increasing the lifetime risk of cancer, intussusception, and gastrointestinal haemorrhage. Routine screening and surveillance are essential to prevent complications from intestinal and extraintestinal malignancies [2].

PJS is extremely rare, with an estimated incidence of one in 8,500 to one in 200,000 births. It results from a germline mutation on chromosome 19p13.3 in the serine/threonine kinase 11 (STK11/LKB1) tumour suppressor gene [3]. The syndrome was first described by Peutz in 1921 in a Dutch family with gastrointestinal polyposis and pigmentations [4].

Diagnostic criteria for Peutz-Jeghers polyps (PJPs) include two or more histologically confirmed PJPs, any number of polyps with a family history of PJS, mucocutaneous pigmentation with a family history of PJS, or polyps in combination with mucocutaneous pigmentation [5].

## Case presentation

**Figure 1:**
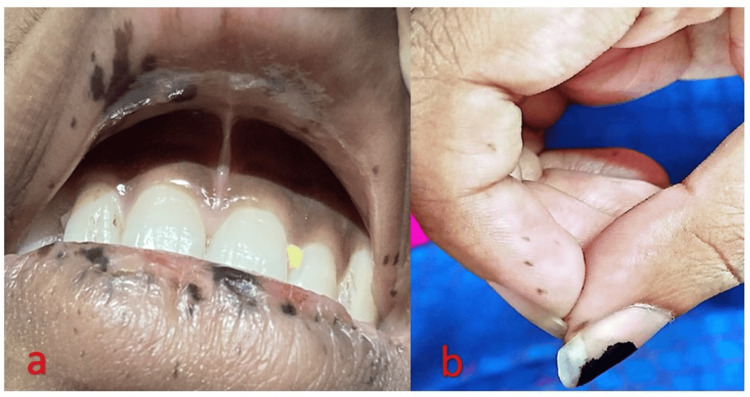
Mucocutaneous pigmentation involving the lips (a) and pulp of the fingers (b).

A 19-year-old female presented with a urinary tract infection and was incidentally found to have anaemia and mucocutaneous pigmentation on the lips, oral cavity, palms, and soles, present since birth. She reported occasional rectal bleeding and intermittent, diffuse abdominal pain but denied significant weight loss, gastrointestinal symptoms, or a family history of PJS. On examination, she was pale, with prominent mucocutaneous pigmentation (Figure 1a, Figure 1b).

Laboratory tests revealed microcytic, hypochromic anaemia with low haemoglobin, MCV, and ferritin, consistent with iron deficiency anaemia, for which she received an iron infusion (Table 1, Table 2).

**Table 1:** Complete blood count during admission CBC: complete blood count; WBC: white blood cells; RBC: red blood cells; MCV: mean corpuscular volume

Variables	Patient values	Normal range
WBC	16 x 10^3^/uL	4.5-10.4 x 10^3^/uL
RBC	4.53 x 10^6^/uL	3.70-5.30 x 10^6^/uL
Hemoglobin	9.7 gm/dL	11.0-16.0 gm/dL
Hematocrit	31.7 %	35.0-47.0%
MCV	70.0 fL	81.0-97.0 fL
MCH	21.4 pg	26-32 pg

**Table 2:** Iron profile in further analysis TIBC: total iron-binding capacity

Variables	Patient values	Normal range
Iron	10 mcg/dL	50-170 mcg/dL
TIBC	348 mcg/dL	250-450 mcg/dL
Ferritin	13 ng/ml	10-120 ng/ml
Iron saturation percentage	2.87 %	20-55%

**Figure 2:**
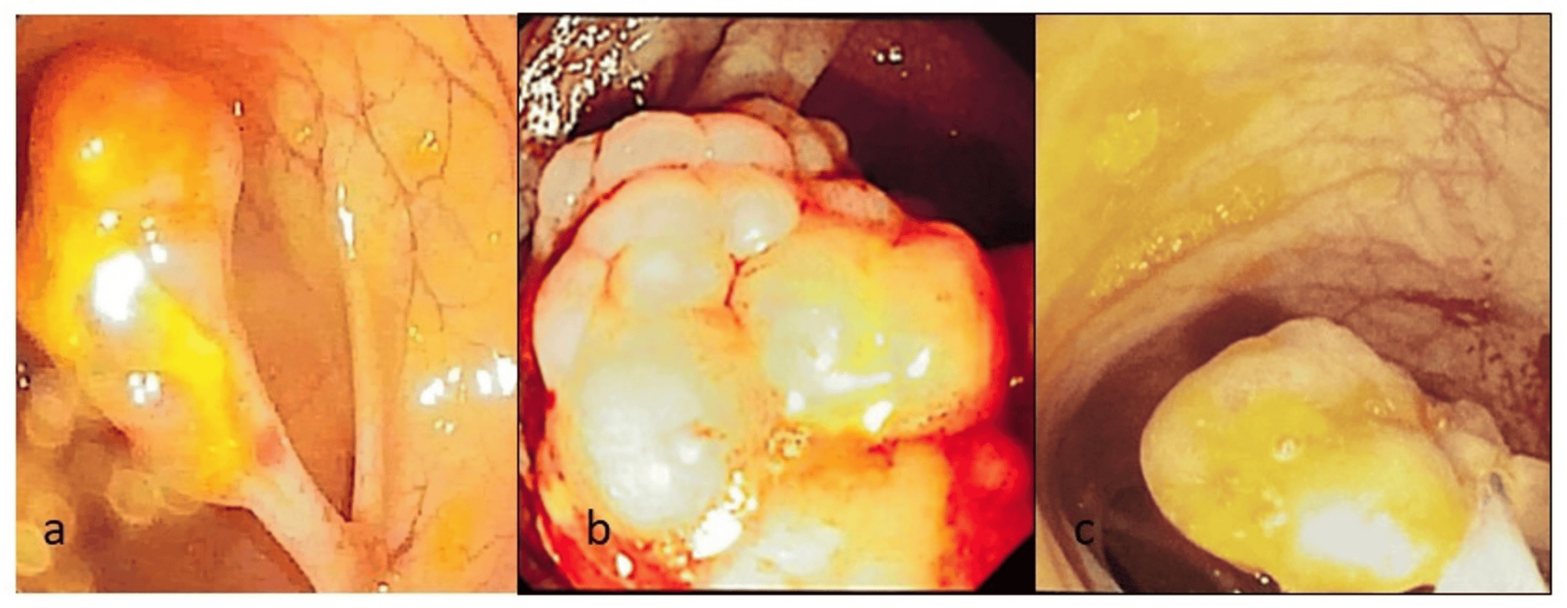
Colonoscopy findings shows a pedunculated polyp in the colon (Figure 2a) and large polypoidal mass within the colon (Figure 2b). Snare polypectomy was performed to remove the polyp (Figure 2c).

Esophagogastroduodenoscopy (EGD) revealed multiple gastric polyps, while colonoscopy showed numerous pedunculated polyps (5-20 mm) throughout the colon (Figure 2).

**Figure 3:**
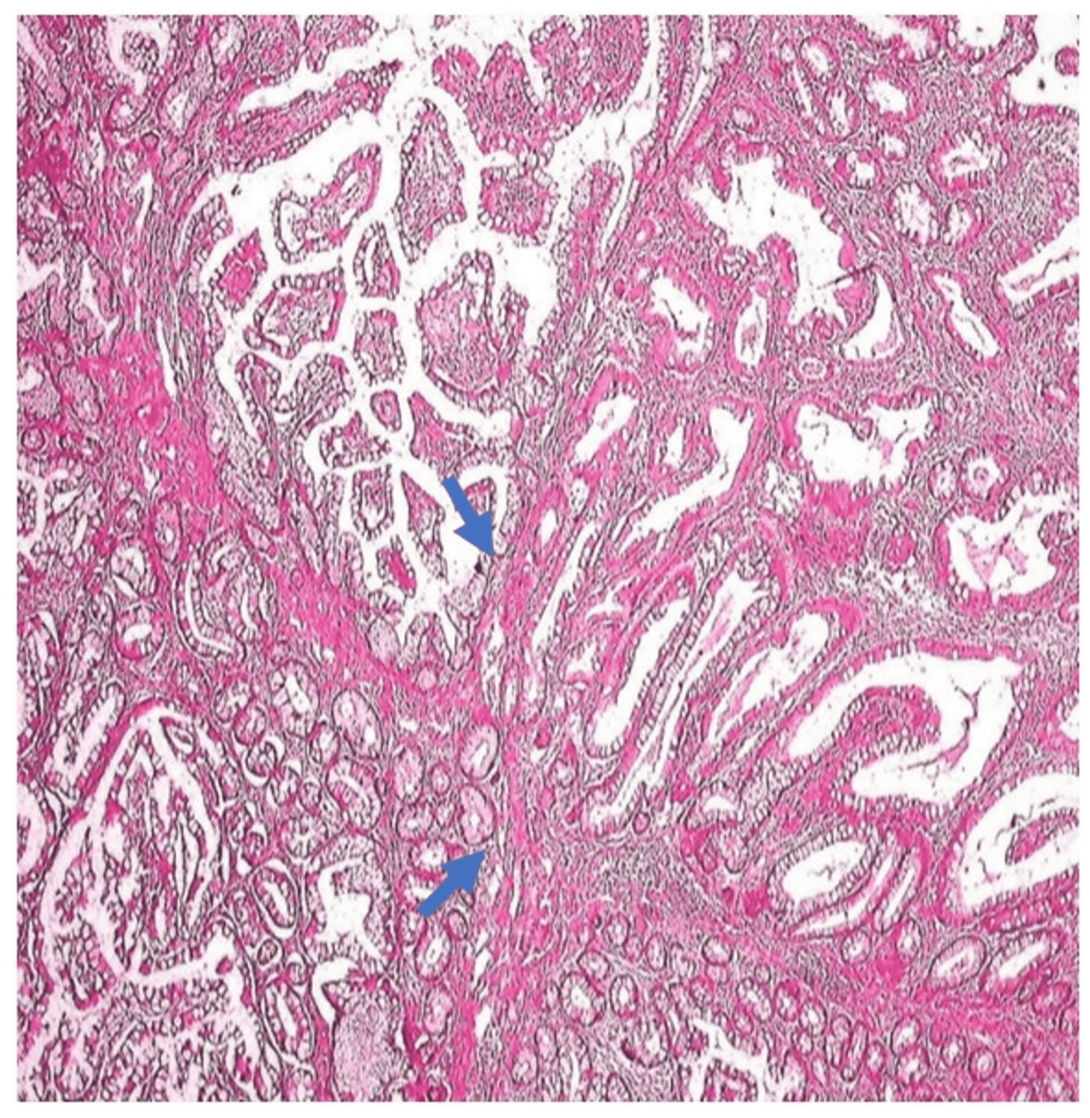
Histological section showing Peutz-Jeghers polyp with arborizing smooth muscle fibers extending from the muscularis mucosa into the lamina propria, forming a tree-like pattern within the colonic epithelium.

Seven large polyps were removed via snare polypectomy and sent for histopathological analysis. Histopathology confirmed hamartomatous polyps with smooth muscle hyperplasia and an arborized growth pattern, consistent with PJS (Figure 3).

**Figure 4:**
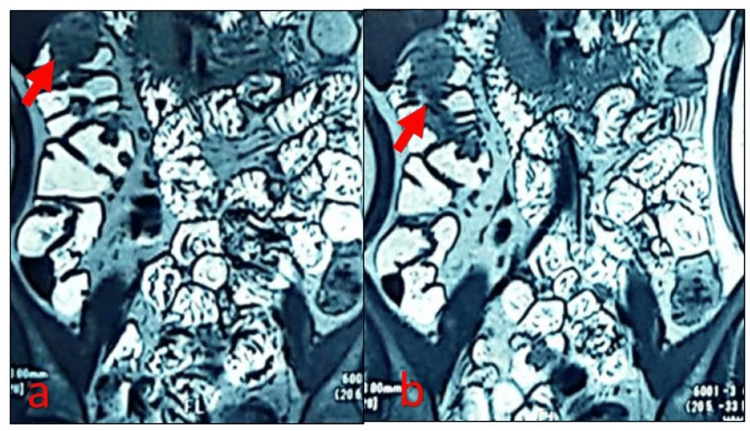
Coronal magnetic resonance enterography (MRE) images show multiple polypoid lesions (marked by red arrows) within the intestine.

Magnetic resonance enterography (MRE) detected multiple polyps in the small and large intestines (Figure 4a, Figure 4b).

During the second session of polypectomy, the patient developed immediate bleeding from the polypectomy site, which was successfully managed with local hemostasis using a hemostatic clip and one unit of blood transfusion. She was referred to gynaecology-oncology for breast, uterine, and ovarian cancer screening and advised to maintain regular gastroenterology follow-up. First-degree relatives were recommended for screening.

## Discussion

Mucocutaneous pigmentation is observed in approximately 95% of PJS patients and often serves as an early clinical clue before the onset of gastrointestinal symptoms. These flat, blue-grey lesions (1-5 mm) are most commonly located on the vermilion border of the lips (94%), followed by the hands (74%), buccal mucosa (64%), and feet (62%). They may also be present in other areas, such as the nostrils, peri-anal region, and internal sites, including the ureter, gallbladder, bladder, and bronchi [6]. Typically present from birth or early childhood, these pigmentations often fade with age, except for those on the buccal mucosa, which tend to persist [7]. The pigmentation is linked to mutations in the STK11 tumour suppressor gene, which affects melanocyte biology, although the precise mechanism remains unclear [8].

Hamartomatous polyps are found in 88% of PJS patients, most commonly in the small intestine (64%), colon (64%), stomach (49%), and rectum (32%). They vary in size from 0.1 to 5 cm and typically appear in numbers ranging from one to 20 per affected segment [9]. Symptoms, including bleeding, anaemia, and abdominal pain, occur in 33% of cases by age 10 and in 50% by age 20. Characteristically, these polyps have a tree-like smooth muscle core covered by normal epithelium, are often pedunculated, and are most frequently located in the jejunum. Small intestinal hamartomas often present as surgical emergencies due to obstruction, intussusception, or GI bleeding. A review of 39 cases identified intussusception as the most common cause and abdominal pain as the leading symptom [10]. Paediatric PJS cases show a higher prevalence of de novo STK11 mutations. In children with iron deficiency anaemia and abdominal pain, thorough examination and family history are crucial for early detection of PJS, enabling timely diagnosis and cancer risk screening [11].

This case is unique for several reasons. The patient has no family history of PJS and presents with gastric polyps in the fundus of the stomach, along with multiple giant polyps in the colon, leading to massive bleeding during polypectomy. Due to the unavailability of genetic testing in our country, we were unable to perform it. A similar case has been reported in which a patient had isolated PJS-type gastric polyps without a relevant family history and also lacked typical mucocutaneous pigmentation [12]. Due to the presence of large polyps, this patient experienced frequent episodes of abdominal pain and per rectal bleeding, leading to anaemia. However, there was no history of acute abdominal events such as intussusception.

PJS increases cancer risk by up to 15 times compared to the general population, with a 132-fold higher risk of pancreatic cancer [9]. The hamartoma-adenoma-carcinoma sequence may cause polyps to become malignant, necessitating thorough evaluation of the gastrointestinal system and other solid tumours [13]. Surveillance in PJS focuses on early carcinoma detection and the removal of large or eroding polyps to prevent complications like bleeding, anaemia, and obstruction [14]. Clinical guidelines recommend annual blood counts and physical exams, with cancer surveillance starting at age eight through upper GI endoscopy, video capsule endoscopy (VCE), and colonoscopy. If polyps are detected, follow-ups should be every two to three years. Additional screenings include abdominal ultrasonography or MRCP/ERCP from age 25, mammograms from age 50, and annual pelvic exams and breast exams starting at age 18. Serial CA-125 testing and endometrial biopsies are recommended for women starting at age 20 [15]. Endoscopic treatment is the main therapy for PJS, with additional laparoscopy or surgery if complications arise or polyps are large. Endoscopic mucosal resection (EMR) is recommended for polyps larger than 2.0 cm or in cases of intussusception or anaemia. Imaging or endoscopic ultrasonography (EUS) can aid in preoperative evaluation. EMR is minimally invasive and serves both diagnostic and therapeutic purposes [16]. Post-polypectomy bleeding is a frequent complication, but it can be effectively controlled using various endoscopic hemostatic techniques. Endoscopists should be skilled in methods such as pressure application, epinephrine injection, cautery, hemoclips, loops, and band ligation to ensure prompt and successful management [17].

This case highlights the importance of gastrointestinal surveillance in young patients presenting with iron deficiency anaemia of unexplained origin, especially when accompanied by mucocutaneous pigmentation, even in the absence of a significant family history.

## Conclusions

PJP should be investigated in young patients with mucocutaneous pigmentation and unexplained iron deficiency anaemia, even without a family history, as de novo mutations can cause PJS. Comprehensive GI evaluation, including upper GI endoscopy, colonoscopy, and small bowel imaging, is essential for diagnosis. The presence of hamartomatous polyps on histopathology, along with mucocutaneous pigmentation or family history, confirms the diagnosis. Management includes iron supplementation and endoscopic polyp removal to reduce the risk of chronic blood loss and malignancy. A multidisciplinary approach involving gastroenterology, gynaecology and obstetrics, oncology, clinical genetics, and psychology ensures early detection, effective surveillance, and improved patient outcomes. Routine screening and regular follow-up are essential for the early detection of tumours, allowing timely intervention to reduce the risk of malignancy.
